# Molecular Diagnostics

**DOI:** 10.1155/2013/387486

**Published:** 2013-09-03

**Authors:** Akanchha Kesari, Ashwin Dalal, Girdhari Lal, Sachchida Nand Pandey

**Affiliations:** ^1^Center for Genetic Medicine Research, Children's National Medical Center, 111 Michigan Avenue, NW, Washington, DC 20010, USA; ^2^Centre for DNA Fingerprinting and Diagnostics, Hyderabad 500001, India; ^3^National Centre for Cell Science, Pune 411007, India

The laboratory medicine is advancing towards the use of molecular techniques and molecular diagnostics for the detection of leukemia, genetic disorders, preimplantation screenings, pharmacogenomics, infectious diseases, and cancers. Molecular diagnostics dates back to 1980s but now has grown exponentially. The field has a wide range of applications including identifying individuals at risk of developing certain disorders (either genetic or nongenetic), screening of apparently healthy populations, determining prognosis, diagnosis, and monitoring patient's response to therapy. Molecular diagnosis is a very helpful tool that helps to provide very fast and accurate information about the heritable defects. This in turn helps the physicians to provide carrier analysis, prenatal counseling to the family members at risk. Personalized medicine allows an individual to have a panel of genetic tests performed to determine predisposition to disease. Molecular diagnostics can also aid in making decision about therapy to be used for genetic disorders. 

In this special issue, we try to bring the new and existing molecular diagnostics techniques available for the diagnosis of heritable, cancerous, and infectious conditions. The papers in the special issue have shown the use of new techniques that can help the laboratory to better serve the patient population in the coming years. This special issue has six articles, where one review article discussed use of proteomics approach for molecular diagnosis of the cancer, followed by an article focusing on cancer diagnosis. These articles describe recent advancement in the field as well as useful tools in the assessment of cancer. One of the papers has efficiently used the fluorescence in situ hybridization (FISH), quantitative reverse transcriptase PCR (qRT-PCR) to track the fusion gene in prostate cancer. 

K. Tuononen et al. have compared four different methods to identify the anaplastic lymphoma kinase (*ALK*) gene rearrangements that occur in a subgroup of non-small cell lung carcinomas (NSCLC). Q. Zou et al. have shown the use of biomarker to monitor progression and prognosis of gallbladder cancer. Furthermore, in this issue G. S. Pandey et al. have described a flow cytometry-based detection of intracellular factor VIII which plays important role in very common genetic disorder, hemophilia A. On the same line a PCR-based approach has been described to identify and monitor the pathogens during the outbreak of infections. 

D. Paul et al. have reviewed and discussed tissue culture-based discovery of potential biomarkers using various mass spectrometry-based proteomic approaches. K. Tuononen et al. screened *ALK* gene fusions in lung carcinomas and compared targeted resequencing results with several other methods such as FISH, immunohistochemical staining and qRT-PCR. The authors showed that resequencing results significantly correlated with other methods and emphasized that targeted resequencing proved to be a more promising method for *ALK* gene fusion detection in NSCLC. Furthermore, targeted sequencing may also help to reduce sample volume and time required for analysis of lung carcinomas.

Q. Zou et al. showed that overexpression of PSCA and Oct-4 in gallbladder adenocarcinomas (GBCs) correlated with decreased patients' survival and proposed an important biomarker for reflecting the carcinogenesis, progression, metastasis or invasive potential and prognosis of gallbladder carcinoma. The authors further suggested that expression levels of PSCA and Oct-4 could be used as biomarker for early detection of GBCs in benign lesions as well as population screening.

A. Fernández-Serra et al. have demonstrated the usefulness of a commercial tricolor probe FISH approach for the identification of the transmembrane-serine protease gene (*TMPRSS2*) in combination with erythroblast transformation-specific (*ETS*) member *ERG* (v-ets erythroblastosis virus E26 oncogene homolog avian) status and provided a genetic mechanism responsible for gene fusion. They have also described the efficient concordance between FISH and qRT-PCR, and have demonstrated that the *TMPRSS2-ERG* is specific for prostate cancer (PCa) but might not be a good prognostic biomarker in case of PCa patients who undergone radical prostatectomy.

Using flow cytometry-based assay, G. S. Pandey et al. detected factor VIII protein in peripheral blood mononuclear cells. The authors described an indirect intracellular staining method using various monoclonal antibodies to different domain of human factor VIII protein. They estimated the protein expression by measuring the mean and median fluorescence intensities (MFI) of monoclonal antibodies and further confirmed flow cytometry data with intracellular staining of transiently transfected cell lines. Furthermore, monoclonal antibodies are able to detect the intracellular factor VIII protein in PBMCs. Together, G. S. Pandey et al. provided rapid and reliable screening methods to detect intracellular factor VIII levels in PBMCs of hemophilia A patients.

Surveillance of infectious agents is one of the best ways to monitor the appearance of infectious disease in the population and provides a very good tool to prompt the regulatory authorities to control the endemics. Prompt and statistically reliable methods to detect such outbreaks require very simple, sensitive, and specific tools and technologies. H. Sugiura et al. used prescription surveillance and PCR-based approach to identify the pathogens during the outbreaks of infection. They analyzed the nationwide common cold prescription data of Japanese surveillance system and also experimentally proved the existence of causative agents such as *Mycobacterium pneumoniae* and respiratory syncytial virus (RSV) during the peak of prescription duration. This provides interesting diagnostic strategies to monitor the presence of infectious agents in the communities.

We hope that papers in this special issue will provide some new insights and contribute to highlight the recent advancement of clinical molecular diagnostics. As tools and technologies improve with time, updates from laboratories across the world will enrich our better understanding, diagnosis, and treatment of patients. 

